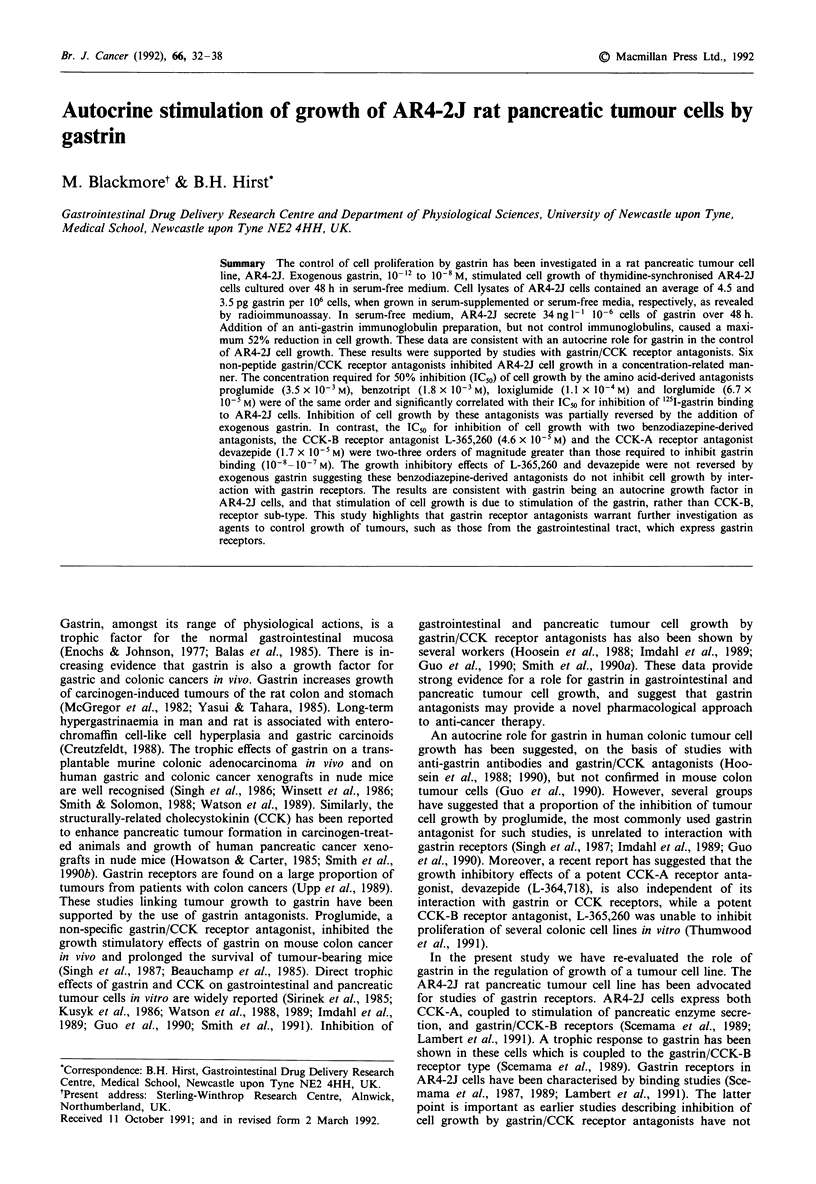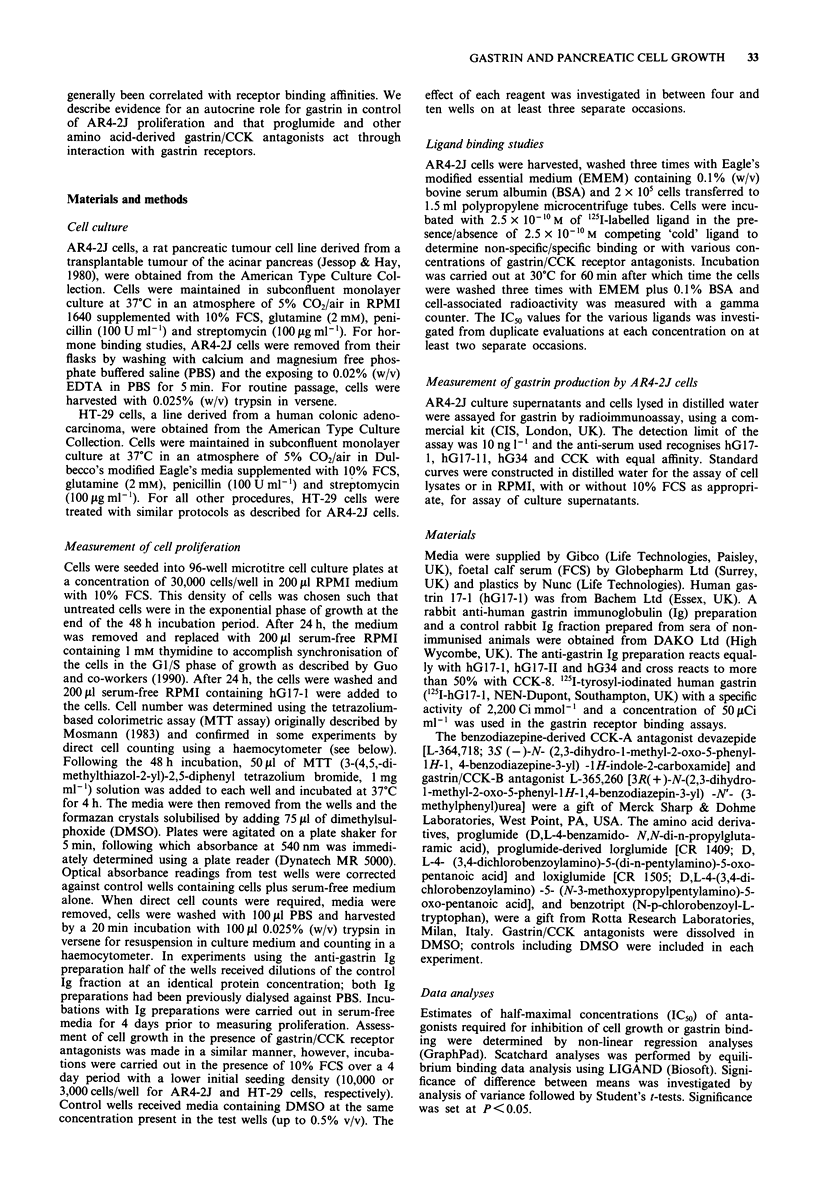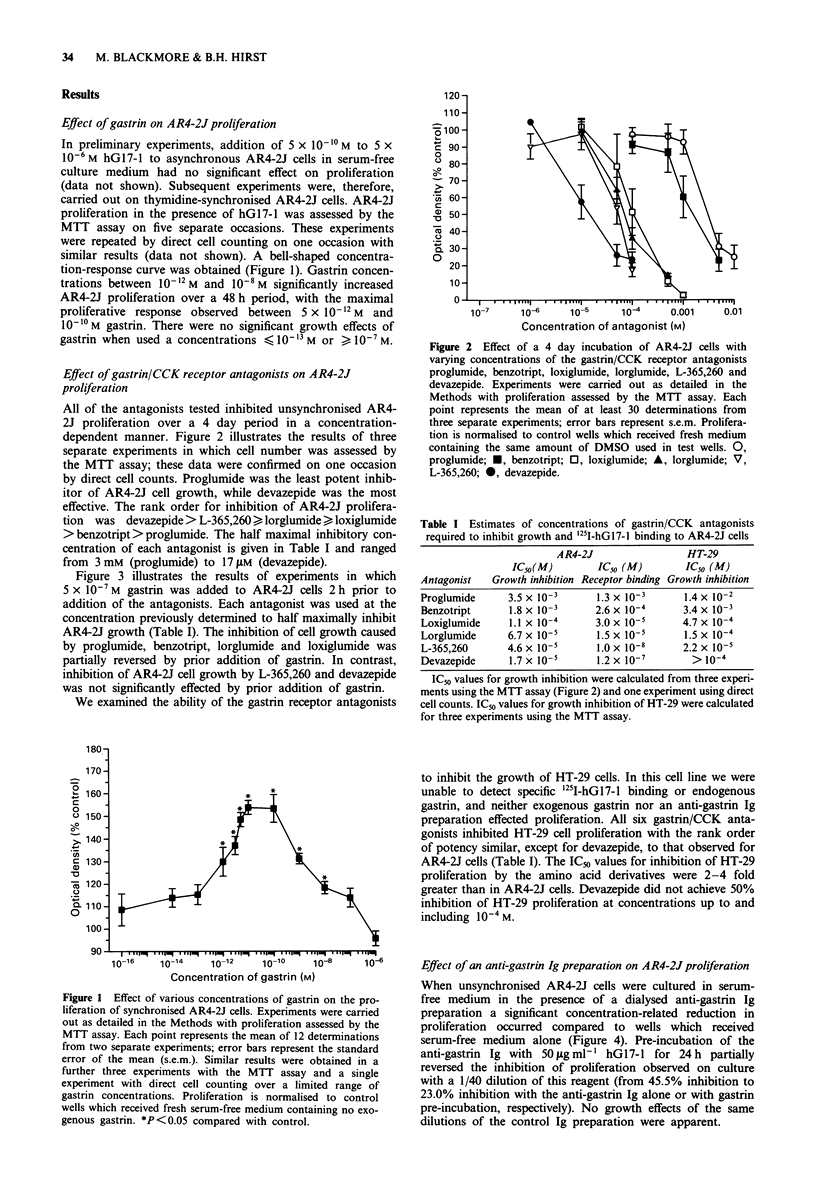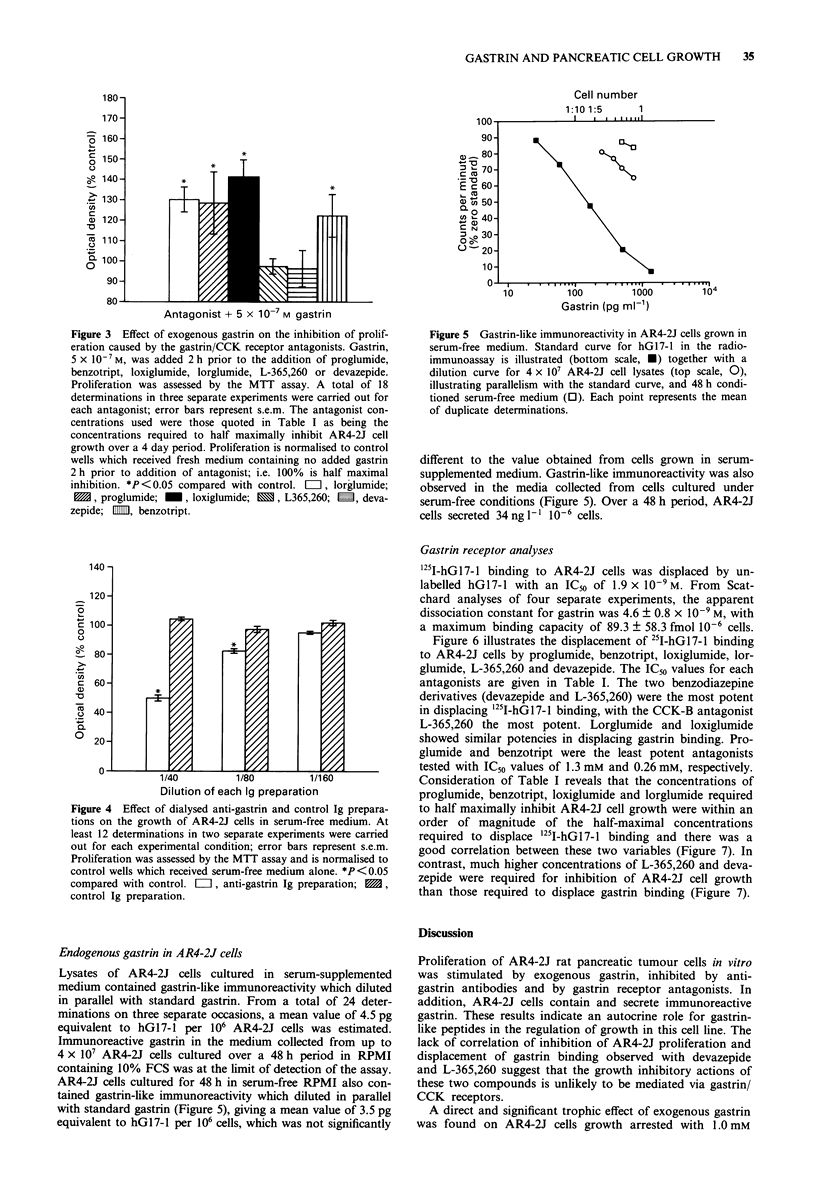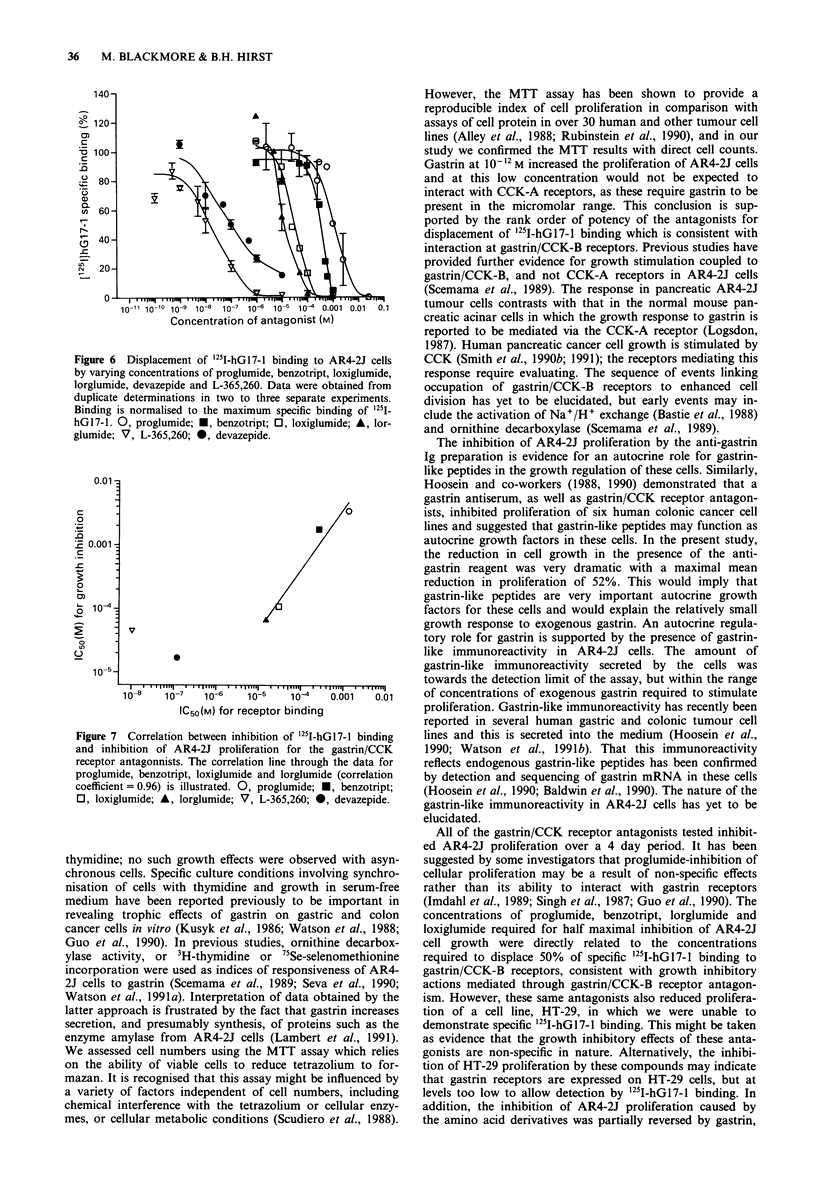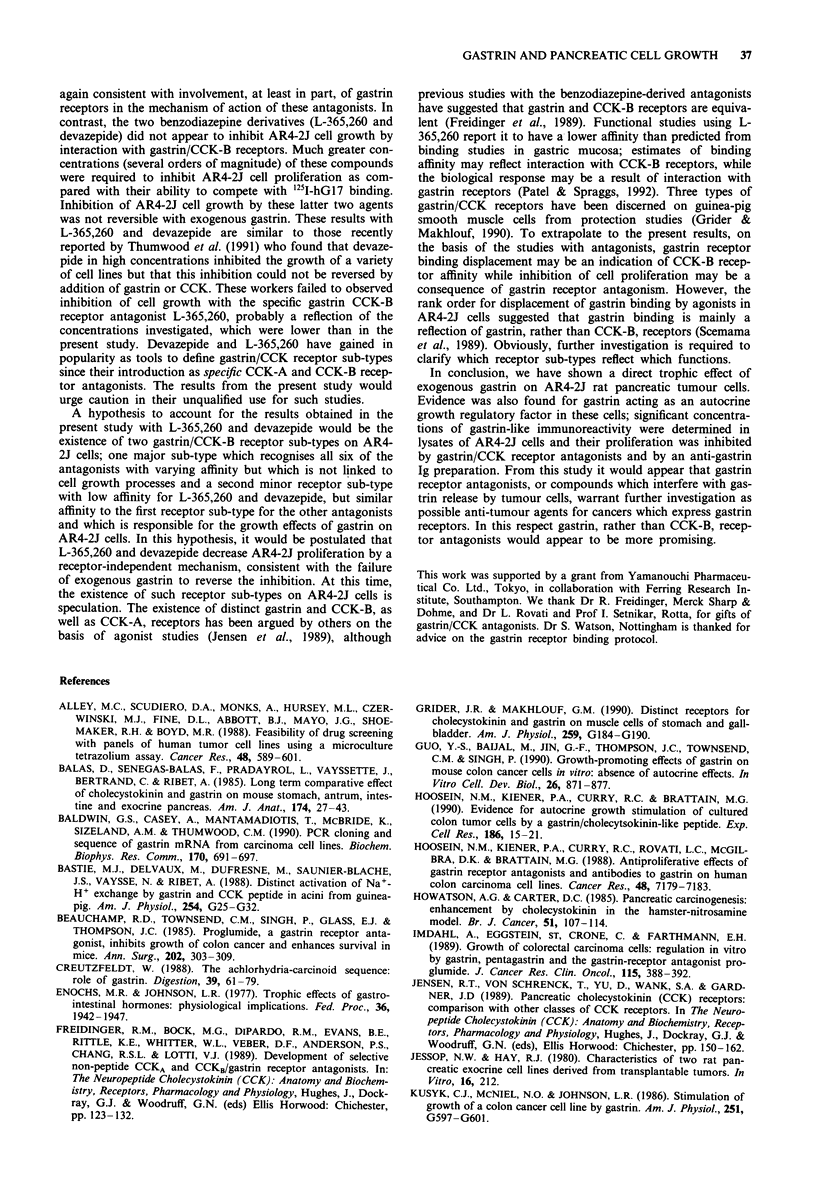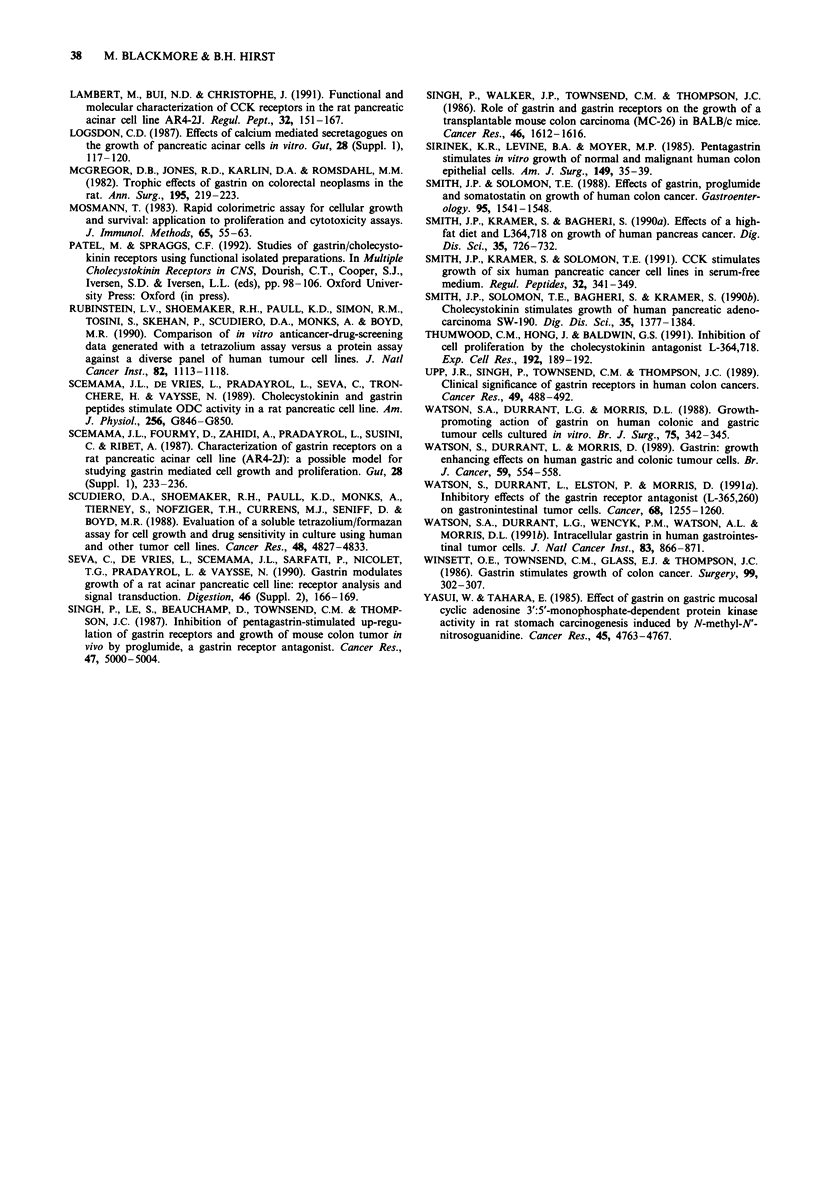# Autocrine stimulation of growth of AR4-2J rat pancreatic tumour cells by gastrin.

**DOI:** 10.1038/bjc.1992.212

**Published:** 1992-07

**Authors:** M. Blackmore, B. H. Hirst

**Affiliations:** Gastrointestinal Drug Delivery Research Centre, University of Newcastle upon Tyne, Medical School, UK.

## Abstract

The control of cell proliferation by gastrin has been investigated in a rat pancreatic tumour cell line, AR4-2J. Exogenous gastrin, 10(-12) to 10(-8) M, stimulated cell growth of thymidine-synchronised AR4-2J cells cultured over 48 h in serum-free medium. Cell lysates of AR4-2J cells contained an average of 4.5 and 3.5 pg gastrin per 10(6) cells, when grown in serum-supplemented or serum-free media, respectively, as revealed by radioimmunoassay. In serum-free medium, AR4-2J secrete 34 ng 1(-1) 10(-6) cells of gastrin over 48 h. Addition of an anti-gastrin immunoglobulin preparation, but not control immunoglobulins, caused a maximum 52% reduction in cell growth. These data are consistent with an autocrine role for gastrin in the control of AR4-2J cell growth. These results were supported by studies with gastrin/CCK receptor antagonists. Six non-peptide gastrin/CCK receptor antagonists inhibited AR4-2J cell growth in a concentration-related manner. The concentration required for 50% inhibition (IC50) of cell growth by the amino acid-derived antagonists proglumide (3.5 x 10(-3) M), benzotript (1.8 x 10(-3) M), loxiglumide (1.1 x 10(-4) M) and lorglumide (6.7 x 10(-5) M) were of the same order and significantly correlated with their IC50 for inhibition of 125I-gastrin binding to AR4-2J cells. Inhibition of cell growth by these antagonists was partially reversed by the addition of exogenous gastrin. In contrast, the IC50 for inhibition of cell growth with two benzodiazepine-derived antagonists, the CCK-B receptor antagonist L-365,260 (4.6 x 10(-5) M) and the CCK-A receptor antagonist devazepide (1.7 x 10(-5) M) were two-three orders of magnitude greater than those required to inhibit gastrin binding (10(-8)-10(-7) M). The growth inhibitory effects of L-365,260 and devazepide were not reversed by exogenous gastrin suggesting these benzodiazepine-derived antagonists do not inhibit cell growth by interaction with gastrin receptors. The results are consistent with gastrin being an autocrine growth factor in AR4-2J cells, and that stimulation of cell growth is due to stimulation of the gastrin, rather than CCK-B, receptor sub-type. This study highlights that gastrin receptor antagonists warrant further investigation as agents to control growth of tumours, such as those from the gastrointestinal tract, which express gastrin receptors.


*Br. J. Cancer* (1992), **66**, 32–38 © Macmillan Press Ltd., 1992

### Autocrine stimulation of growth of AR4-2J rat pancreatic tumour cells by gastrin

M. Blackmore[†] & B.H. Hirst[*]

*Gastrointestinal Drug Delivery Research Centre and Department of Physiological Sciences, University of Newcastle upon Tyne, Medical School, Newcastle upon Tyne NE2 4HH, UK.*

**Summary** The control of cell proliferation by gastrin has been investigated in a rat pancreatic tumour cell line, AR4-2J. Exogenous gastrin, $10^{-12}$ to $10^{-8}$ M, stimulated cell growth of thymidine-synchronised AR4-2J cells cultured over 48 h in serum-free medium. Cell lysates of AR4-2J cells contained an average of 4.5 and 3.5 pg gastrin per $10^6$ cells, when grown in serum-supplemented or serum-free media, respectively, as revealed by radioimmunoassay. In serum-free medium, AR4-2J secrete 34 ng $l^{-1}$ $10^{-6}$ cells of gastrin over 48 h. Addition of an anti-gastrin immunoglobulin preparation, but not control immunoglobulins, caused a maximum 52% reduction in cell growth. These data are consistent with an autocrine role for gastrin in the control of AR4-2J cell growth. These results were supported by studies with gastrin/CCK receptor antagonists. Six non-peptide gastrin/CCK receptor antagonists inhibited AR4-2J cell growth in a concentration-related manner. The concentration required for 50% inhibition ($IC_{50}$) of cell growth by the amino acid-derived antagonists proglumide ($3.5 \times 10^{-3}$ M), benzotript ($1.8 \times 10^{-3}$ M), loxiglumide ($1.1 \times 10^{-4}$ M) and lorglumide ($6.7 \times 10^{-5}$ M) were of the same order and significantly correlated with their $IC_{50}$ for inhibition of $^{125}$I-gastrin binding to AR4-2J cells. Inhibition of cell growth by these antagonists was partially reversed by the addition of exogenous gastrin. In contrast, the $IC_{50}$ for inhibition of cell growth with two benzodiazepine-derived antagonists, the CCK-B receptor antagonist L-365,260 ($4.6 \times 10^{-5}$ M) and the CCK-A receptor antagonist devazepide ($1.7 \times 10^{-5}$ M) were two-three orders of magnitude greater than those required to inhibit gastrin binding ($10^{-8}$–$10^{-7}$ M). The growth inhibitory effects of L-365,260 and devazepide were not reversed by exogenous gastrin suggesting these benzodiazepine-derived antagonists do not inhibit cell growth by interaction with gastrin receptors. The results are consistent with gastrin being an autocrine growth factor in AR4-2J cells, and that stimulation of cell growth is due to stimulation of the gastrin, rather than CCK-B, receptor sub-type. This study highlights that gastrin receptor antagonists warrant further investigation as agents to control growth of tumours, such as those from the gastrointestinal tract, which express gastrin receptors.

Gastrin, amongst its range of physiological actions, is a trophic factor for the normal gastrointestinal mucosa (Enochs & Johnson, 1977; Balas *et al.*, 1985). There is increasing evidence that gastrin is also a growth factor for gastric and colonic cancers *in vivo*. Gastrin increases growth of carcinogen-induced tumours of the rat colon and stomach (McGregor *et al.*, 1982; Yasui & Tahara, 1985). Long-term hypergastrinaemia in man and rat is associated with enterochromaffin cell-like cell hyperplasia and gastric carcinoids (Creutzfeldt, 1988). The trophic effects of gastrin on a transplantable murine colonic adenocarcinoma *in vivo* and on human gastric and colonic cancer xenografts in nude mice are well recognised (Singh *et al.*, 1986; Winsett *et al.*, 1986; Smith & Solomon, 1988; Watson *et al.*, 1989). Similarly, the structurally-related cholecystokinin (CCK) has been reported to enhance pancreatic tumour formation in carcinogen-treated animals and growth of human pancreatic cancer xenografts in nude mice (Howatson & Carter, 1985; Smith *et al.*, 1990*b*). Gastrin receptors are found on a large proportion of tumours from patients with colon cancers (Upp *et al.*, 1989). These studies linking tumour growth to gastrin have been supported by the use of gastrin antagonists. Proglumide, a non-specific gastrin/CCK receptor antagonist, inhibited the growth stimulatory effects of gastrin on mouse colon cancer *in vivo* and prolonged the survival of tumour-bearing mice (Singh *et al.*, 1987; Beauchamp *et al.*, 1985). Direct trophic effects of gastrin and CCK on gastrointestinal and pancreatic tumour cells *in vitro* are widely reported (Sirinek *et al.*, 1985; Kusyk *et al.*, 1986; Watson *et al.*, 1988, 1989; Imdahl *et al.*, 1989; Guo *et al.*, 1990; Smith *et al.*, 1991). Inhibition of gastrointestinal and pancreatic tumour cell growth by gastrin/CCK receptor antagonists has also been shown by several workers (Hoosein *et al.*, 1988; Imdahl *et al.*, 1989; Guo *et al.*, 1990; Smith *et al.*, 1990*a*). These data provide strong evidence for a role for gastrin in gastrointestinal and pancreatic tumour cell growth, and suggest that gastrin antagonists may provide a novel pharmacological approach to anti-cancer therapy.

An autocrine role for gastrin in human colonic tumour cell growth has been suggested, on the basis of studies with anti-gastrin antibodies and gastrin/CCK antagonists (Hoosein *et al.*, 1988; 1990), but not confirmed in mouse colon tumour cells (Guo *et al.*, 1990). However, several groups have suggested that a proportion of the inhibition of tumour cell growth by proglumide, the most commonly used gastrin antagonist for such studies, is unrelated to interaction with gastrin receptors (Singh *et al.*, 1987; Imdahl *et al.*, 1989; Guo *et al.*, 1990). Moreover, a recent report has suggested that the growth inhibitory effects of a potent CCK-A receptor antagonist, devazepide (L-364,718), is also independent of its interaction with gastrin or CCK receptors, while a potent CCK-B receptor antagonist, L-365,260 was unable to inhibit proliferation of several colonic cell lines *in vitro* (Thumwood *et al.*, 1991).

In the present study we have re-evaluated the role of gastrin in the regulation of growth of a tumour cell line. The AR4-2J rat pancreatic tumour cell line has been advocated for studies of gastrin receptors. AR4-2J cells express both CCK-A, coupled to stimulation of pancreatic enzyme secretion, and gastrin/CCK-B receptors (Scemama *et al.*, 1989; Lambert *et al.*, 1991). A trophic response to gastrin has been shown in these cells which is coupled to the gastrin/CCK-B receptor type (Scemama *et al.*, 1989). Gastrin receptors in AR4-2J cells have been characterised by binding studies (Scemama *et al.*, 1987, 1989; Lambert *et al.*, 1991). The latter point is important as earlier studies describing inhibition of cell growth by gastrin/CCK receptor antagonists have not

[*]Correspondence: B.H. Hirst, Gastrointestinal Drug Delivery Research Centre, Medical School, Newcastle upon Tyne NE2 4HH, UK.
[†]Present address: Sterling-Winthrop Research Centre, Alnwick, Northumberland, UK.
Received 11 October 1991; and in revised form 2 March 1992.

generally been correlated with receptor binding affinities. We describe evidence for an autocrine role for gastrin in control of AR4-2J proliferation and that proglumide and other amino acid-derived gastrin/CCK antagonists act through interaction with gastrin receptors.

### Materials and methods

### *Cell culture*

AR4-2J cells, a rat pancreatic tumour cell line derived from a transplantable tumour of the acinar pancreas (Jessop & Hay, 1980), were obtained from the American Type Culture Collection. Cells were maintained in subconfluent monolayer culture at 37°C in an atmosphere of 5% $CO_2$/air in RPMI 1640 supplemented with 10% FCS, glutamine (2 mM), penicillin (100 U $ml^{-1}$) and streptomycin (100 $\mu g$ $ml^{-1}$). For hormone binding studies, AR4-2J cells were removed from their flasks by washing with calcium and magnesium free phosphate buffered saline (PBS) and the exposing to 0.02% (w/v) EDTA in PBS for 5 min. For routine passage, cells were harvested with 0.025% (w/v) trypsin in versene.

HT-29 cells, a line derived from a human colonic adenocarcinoma, were obtained from the American Type Culture Collection. Cells were maintained in subconfluent monolayer culture at 37°C in an atmosphere of 5% $CO_2$/air in Dulbecco's modified Eagle's media supplemented with 10% FCS, glutamine (2 mM), penicillin (100 U $ml^{-1}$) and streptomycin (100 $\mu g$ $ml^{-1}$). For all other procedures, HT-29 cells were treated with similar protocols as described for AR4-2J cells.

### *Measurement of cell proliferation*

Cells were seeded into 96-well microtitre cell culture plates at a concentration of 30,000 cells/well in 200 $\mu l$ RPMI medium with 10% FCS. This density of cells was chosen such that untreated cells were in the exponential phase of growth at the end of the 48 h incubation period. After 24 h, the medium was removed and replaced with 200 $\mu l$ serum-free RPMI containing 1 mM thymidine to accomplish synchronisation of the cells in the G1/S phase of growth as described by Guo and co-workers (1990). After 24 h, the cells were washed and 200 $\mu l$ serum-free RPMI containing hG17-1 were added to the cells. Cell number was determined using the tetrazolium-based colorimetric assay (MTT assay) originally described by Mosmann (1983) and confirmed in some experiments by direct cell counting using a haemocytometer (see below). Following the 48 h incubation, 50 $\mu l$ of MTT (3-(4,5,-dimethylthiazol-2-yl)-2,5-diphenyl tetrazolium bromide, 1 mg $ml^{-1}$) solution was added to each well and incubated at 37°C for 4 h. The media were then removed from the wells and the formazan crystals solubilised by adding 75 $\mu l$ of dimethylsulphoxide (DMSO). Plates were agitated on a plate shaker for 5 min, following which absorbance at 540 nm was immediately determined using a plate reader (Dynatech MR 5000). Optical absorbance readings from test wells were corrected against control wells containing cells plus serum-free medium alone. When direct cell counts were required, media were removed, cells were washed with 100 $\mu l$ PBS and harvested by a 20 min incubation with 100 $\mu l$ 0.025% (w/v) trypsin in versene for resuspension in culture medium and counting in a haemocytometer. In experiments using the anti-gastrin Ig preparation half of the wells received dilutions of the control Ig fraction at an identical protein concentration; both Ig preparations had been previously dialysed against PBS. Incubations with Ig preparations were carried out in serum-free media for 4 days prior to measuring proliferation. Assessment of cell growth in the presence of gastrin/CCK receptor antagonists was made in a similar manner, however, incubations were carried out in the presence of 10% FCS over a 4 day period with a lower initial seeding density (10,000 or 3,000 cells/well for AR4-2J and HT-29 cells, respectively). Control wells received media containing DMSO at the same concentration present in the test wells (up to 0.5% v/v). The effect of each reagent was investigated in between four and ten wells on at least three separate occasions.

### *Ligand binding studies*

AR4-2J cells were harvested, washed three times with Eagle's modified essential medium (EMEM) containing 0.1% (w/v) bovine serum albumin (BSA) and $2 \times 10^5$ cells transferred to 1.5 ml polypropylene microcentrifuge tubes. Cells were incubated with $2.5 \times 10^{-10}$ M of $^{125}$I-labelled ligand in the presence/absence of $2.5 \times 10^{-10}$ M competing 'cold' ligand to determine non-specific/specific binding or with various concentrations of gastrin/CCK receptor antagonists. Incubation was carried out at 30°C for 60 min after which time the cells were washed three times with EMEM plus 0.1% BSA and cell-associated radioactivity was measured with a gamma counter. The $IC_{50}$ values for the various ligands was investigated from duplicate evaluations at each concentration on at least two separate occasions.

### *Measurement of gastrin production by AR4-2J cells*

AR4-2J culture supernatants and cells lysed in distilled water were assayed for gastrin by radioimmunoassay, using a commercial kit (CIS, London, UK). The detection limit of the assay was 10 ng $l^{-1}$ and the anti-serum used recognises hG17-1, hG17-11, hG34 and CCK with equal affinity. Standard curves were constructed in distilled water for the assay of cell lysates or in RPMI, with or without 10% FCS as appropriate, for assay of culture supernatants.

### *Materials*

Media were supplied by Gibco (Life Technologies, Paisley, UK), foetal calf serum (FCS) by Globepharm Ltd (Surrey, UK) and plastics by Nunc (Life Technologies). Human gastrin 17-1 (hG17-1) was from Bachem Ltd (Essex, UK). A rabbit anti-human gastrin immunoglobulin (Ig) preparation and a control rabbit Ig fraction prepared from sera of non-immunised animals were obtained from DAKO Ltd (High Wycombe, UK). The anti-gastrin Ig preparation reacts equally with hG17-1, hG17-II and hG34 and cross reacts to more than 50% with CCK-8. $^{125}$I-tyrosyl-iodinated human gastrin ($^{125}$I-hG17-1, NEN-Dupont, Southampton, UK) with a specific activity of 2,200 Ci $mmol^{-1}$ and a concentration of 50 $\mu Ci$ $ml^{-1}$ was used in the gastrin receptor binding assays.

The benzodiazepine-derived CCK-A antagonist devazepide [L-364,718; 3*S* (−)-*N*- (2,3-dihydro-1-methyl-2-oxo-5-phenyl-1*H*-1, 4-benzodiazepine-3-yl) -1*H*-indole-2-carboxamide] and gastrin/CCK-B antagonist L-365,260 [3*R*(+)-*N*-(2,3-dihydro-1-methyl-2-oxo-5-phenyl-1*H*-1,4-benzodiazepin-3-yl) -*N*′- (3-methylphenyl)urea] were a gift of Merck Sharp & Dohme Laboratories, West Point, PA, USA. The amino acid derivatives, proglumide (D,L-4-benzamido- *N*,*N*-di-n-propylglutaramic acid), proglumide-derived lorglumide [CR 1409; D, L-4- (3,4-dichlorobenzoylamino)-5-(di-n-pentylamino)-5-oxo-pentanoic acid] and loxiglumide [CR 1505; D,L-4-(3,4-dichlorobenzoylamino) -5- (*N*-3-methoxypropylpentylamino)-5-oxo-pentanoic acid], and benzotript (N-p-chlorobenzoyl-L-tryptophan), were a gift from Rotta Research Laboratories, Milan, Italy. Gastrin/CCK antagonists were dissolved in DMSO; controls including DMSO were included in each experiment.

### *Data analyses*

Estimates of half-maximal concentrations ($IC_{50}$) of antagonists required for inhibition of cell growth or gastrin binding were determined by non-linear regression analyses (GraphPad). Scatchard analyses was performed by equilibrium binding data analysis using LIGAND (Biosoft). Significance of difference between means was investigated by analysis of variance followed by Student's *t*-tests. Significance was set at $P < 0.05$.

### Results

### *Effect of gastrin on AR4-2J proliferation*

In preliminary experiments, addition of $5 \times 10^{-10}$ M to $5 \times 10^{-6}$ M hG17-1 to asynchronous AR4-2J cells in serum-free culture medium had no significant effect on proliferation (data not shown). Subsequent experiments were, therefore, carried out on thymidine-synchronised AR4-2J cells. AR4-2J proliferation in the presence of hG17-1 was assessed by the MTT assay on five separate occasions. These experiments were repeated by direct cell counting on one occasion with similar results (data not shown). A bell-shaped concentration-response curve was obtained (Figure 1). Gastrin concentrations between $10^{-12}$ M and $10^{-8}$ M significantly increased AR4-2J proliferation over a 48 h period, with the maximal proliferative response observed between $5 \times 10^{-12}$ M and $10^{-10}$ M gastrin. There were no significant growth effects of gastrin when used a concentrations $\leqslant 10^{-13}$ M or $\geqslant 10^{-7}$ M.

### *Effect of gastrin/CCK receptor antagonists on AR4-2J proliferation*

All of the antagonists tested inhibited unsynchronised AR4-2J proliferation over a 4 day period in a concentration-dependent manner. Figure 2 illustrates the results of three separate experiments in which cell number was assessed by the MTT assay; these data were confirmed on one occasion by direct cell counts. Proglumide was the least potent inhibitor of AR4-2J cell growth, while devazepide was the most effective. The rank order for inhibition of AR4-2J proliferation was devazepide > L-365,260 ⩾ lorglumide ⩾ loxiglumide > benzotript > proglumide. The half maximal inhibitory concentration of each antagonist is given in Table I and ranged from 3 mM (proglumide) to 17 μM (devazepide).

Figure 3 illustrates the results of experiments in which $5 \times 10^{-7}$ M gastrin was added to AR4-2J cells 2 h prior to addition of the antagonists. Each antagonist was used at the concentration previously determined to half maximally inhibit AR4-2J growth (Table I). The inhibition of cell growth caused by proglumide, benzotript, lorglumide and loxiglumide was partially reversed by prior addition of gastrin. In contrast, inhibition of AR4-2J cell growth by L-365,260 and devazepide was not significantly effected by prior addition of gastrin.

We examined the ability of the gastrin receptor antagonists to inhibit the growth of HT-29 cells. In this cell line we were unable to detect specific $^{125}$I-hG17-1 binding or endogenous gastrin, and neither exogenous gastrin nor an anti-gastrin Ig preparation effected proliferation. All six gastrin/CCK antagonists inhibited HT-29 cell proliferation with the rank order of potency similar, except for devazepide, to that observed for AR4-2J cells (Table I). The $IC_{50}$ values for inhibition of HT-29 proliferation by the amino acid derivatives were 2–4 fold greater than in AR4-2J cells. Devazepide did not achieve 50% inhibition of HT-29 proliferation at concentrations up to and including $10^{-4}$ M.

**Figure 2** Effect of a 4 day incubation of AR4-2J cells with varying concentrations of the gastrin/CCK receptor antagonists proglumide, benzotript, loxiglumide, lorglumide, L-365,260 and devazepide. Experiments were carried out as detailed in the Methods with proliferation assessed by the MTT assay. Each point represents the mean of at least 30 determinations from three separate experiments; error bars represent s.e.m. Proliferation is normalised to control wells which received fresh medium containing the same amount of DMSO used in test wells. ○, proglumide; ■, benzotript; □, loxiglumide; ▲, lorglumide; ▽, L-365,260; ●, devazepide.

**Table I** Estimates of concentrations of gastrin/CCK antagonists required to inhibit growth and $^{125}$I-hG17-1 binding to AR4-2J cells

| | *AR4-2J* | | *HT-29* |
|---|---|---|---|
| *Antagonist* | *$IC_{50}$(M) Growth inhibition* | *$IC_{50}$ (M) Receptor binding* | *$IC_{50}$ (M) Growth inhibition* |
| Proglumide | $3.5 \times 10^{-3}$ | $1.3 \times 10^{-3}$ | $1.4 \times 10^{-2}$ |
| Benzotript | $1.8 \times 10^{-3}$ | $2.6 \times 10^{-4}$ | $3.4 \times 10^{-3}$ |
| Loxiglumide | $1.1 \times 10^{-4}$ | $3.0 \times 10^{-5}$ | $4.7 \times 10^{-4}$ |
| Lorglumide | $6.7 \times 10^{-5}$ | $1.5 \times 10^{-5}$ | $1.5 \times 10^{-4}$ |
| L-365,260 | $4.6 \times 10^{-5}$ | $1.0 \times 10^{-8}$ | $2.2 \times 10^{-5}$ |
| Devazepide | $1.7 \times 10^{-5}$ | $1.2 \times 10^{-7}$ | $> 10^{-4}$ |

$IC_{50}$ values for growth inhibition were calculated from three experiments using the MTT assay (Figure 2) and one experiment using direct cell counts. $IC_{50}$ values for growth inhibition of HT-29 were calculated for three experiments using the MTT assay.

**Figure 1** Effect of various concentrations of gastrin on the proliferation of synchronised AR4-2J cells. Experiments were carried out as detailed in the Methods with proliferation assessed by the MTT assay. Each point represents the mean of 12 determinations from two separate experiments; error bars represent the standard error of the mean (s.e.m.). Similar results were obtained in a further three experiments with the MTT assay and a single experiment with direct cell counting over a limited range of gastrin concentrations. Proliferation is normalised to control wells which received fresh serum-free medium containing no exogenous gastrin. *$P < 0.05$ compared with control.

### *Effect of an anti-gastrin Ig preparation on AR4-2J proliferation*

When unsynchronised AR4-2J cells were cultured in serum-free medium in the presence of a dialysed anti-gastrin Ig preparation a significant concentration-related reduction in proliferation occurred compared to wells which received serum-free medium alone (Figure 4). Pre-incubation of the anti-gastrin Ig with 50 μg ml$^{-1}$ hG17-1 for 24 h partially reversed the inhibition of proliferation observed on culture with a 1/40 dilution of this reagent (from 45.5% inhibition to 23.0% inhibition with the anti-gastrin Ig alone or with gastrin pre-incubation, respectively). No growth effects of the same dilutions of the control Ig preparation were apparent.

**Figure 3** Effect of exogenous gastrin on the inhibition of proliferation caused by the gastrin/CCK receptor antagonists. Gastrin, $5 \times 10^{-7}$ M, was added 2 h prior to the addition of proglumide, benzotript, loxiglumide, lorglumide, L-365,260 or devazepide. Proliferation was assessed by the MTT assay. A total of 18 determinations in three separate experiments were carried out for each antagonist; error bars represent s.e.m. The antagonist concentrations used were those quoted in Table I as being the concentrations required to half maximally inhibit AR4-2J cell growth over a 4 day period. Proliferation is normalised to control wells which received fresh medium containing no added gastrin 2 h prior to addition of antagonist; i.e. 100% is half maximal inhibition. *$P<0.05$ compared with control. □, lorglumide; ▨, proglumide; ■, loxiglumide; ▧, L365,260; ▤, devazepide; ▥, benzotript.

**Figure 4** Effect of dialysed anti-gastrin and control Ig preparations on the growth of AR4-2J cells in serum-free medium. At least 12 determinations in two separate experiments were carried out for each experimental condition; error bars represent s.e.m. Proliferation was assessed by the MTT assay and is normalised to control wells which received serum-free medium alone. *$P<0.05$ compared with control. □, anti-gastrin Ig preparation; ▨, control Ig preparation.

*Endogenous gastrin in AR4-2J cells*

Lysates of AR4-2J cells cultured in serum-supplemented medium contained gastrin-like immunoreactivity which diluted in parallel with standard gastrin. From a total of 24 determinations on three separate occasions, a mean value of 4.5 pg equivalent to hG17-1 per $10^6$ AR4-2J cells was estimated. Immunoreactive gastrin in the medium collected from up to $4 \times 10^7$ AR4-2J cells cultured over a 48 h period in RPMI containing 10% FCS was at the limit of detection of the assay. AR4-2J cells cultured for 48 h in serum-free RPMI also contained gastrin-like immunoreactivity which diluted in parallel with standard gastrin (Figure 5), giving a mean value of 3.5 pg equivalent to hG17-1 per $10^6$ cells, which was not significantly different to the value obtained from cells grown in serum-supplemented medium. Gastrin-like immunoreactivity was also observed in the media collected from cells cultured under serum-free conditions (Figure 5). Over a 48 h period, AR4-2J cells secreted 34 ng $l^{-1}$ $10^{-6}$ cells.

**Figure 5** Gastrin-like immunoreactivity in AR4-2J cells grown in serum-free medium. Standard curve for hG17-1 in the radioimmunoassay is illustrated (bottom scale, ■) together with a dilution curve for $4 \times 10^7$ AR4-2J cell lysates (top scale, O), illustrating parallelism with the standard curve, and 48 h conditioned serum-free medium (□). Each point represents the mean of duplicate determinations.

*Gastrin receptor analyses*

$^{125}$I-hG17-1 binding to AR4-2J cells was displaced by unlabelled hG17-1 with an $IC_{50}$ of $1.9 \times 10^{-9}$ M. From Scatchard analyses of four separate experiments, the apparent dissociation constant for gastrin was $4.6 \pm 0.8 \times 10^{-9}$ M, with a maximum binding capacity of $89.3 \pm 58.3$ fmol $10^{-6}$ cells.

Figure 6 illustrates the displacement of $^{25}$I-hG17-1 binding to AR4-2J cells by proglumide, benzotript, loxiglumide, lorglumide, L-365,260 and devazepide. The $IC_{50}$ values for each antagonists are given in Table I. The two benzodiazepine derivatives (devazepide and L-365,260) were the most potent in displacing $^{125}$I-hG17-1 binding, with the CCK-B antagonist L-365,260 the most potent. Lorglumide and loxiglumide showed similar potencies in displacing gastrin binding. Proglumide and benzotript were the least potent antagonists tested with $IC_{50}$ values of 1.3 mM and 0.26 mM, respectively. Consideration of Table I reveals that the concentrations of proglumide, benzotript, loxiglumide and lorglumide required to half maximally inhibit AR4-2J cell growth were within an order of magnitude of the half-maximal concentrations required to displace $^{125}$I-hG17-1 binding and there was a good correlation between these two variables (Figure 7). In contrast, much higher concentrations of L-365,260 and devazepide were required for inhibition of AR4-2J cell growth than those required to displace gastrin binding (Figure 7).

### Discussion

Proliferation of AR4-2J rat pancreatic tumour cells *in vitro* was stimulated by exogenous gastrin, inhibited by anti-gastrin antibodies and by gastrin receptor antagonists. In addition, AR4-2J cells contain and secrete immunoreactive gastrin. These results indicate an autocrine role for gastrin-like peptides in the regulation of growth in this cell line. The lack of correlation of inhibition of AR4-2J proliferation and displacement of gastrin binding observed with devazepide and L-365,260 suggest that the growth inhibitory actions of these two compounds is unlikely to be mediated via gastrin/CCK receptors.

A direct and significant trophic effect of exogenous gastrin was found on AR4-2J cells growth arrested with 1.0 mM

**Figure 6** Displacement of $^{125}I$-hG17-1 binding to AR4-2J cells by varying concentrations of proglumide, benzotript, loxiglumide, lorglumide, devazepide and L-365,260. Data were obtained from duplicate determinations in two to three separate experiments. Binding is normalised to the maximum specific binding of $^{125}I$-hG17-1. O, proglumide; ■, benzotript; □, loxiglumide; ▲, lorglumide; ∇, L-365,260; ●, devazepide.

**Figure 7** Correlation between inhibition of $^{125}I$-hG17-1 binding and inhibition of AR4-2J proliferation for the gastrin/CCK receptor antagonnists. The correlation line through the data for proglumide, benzotript, loxiglumide and lorglumide (correlation coefficient = 0.96) is illustrated. O, proglumide; ■, benzotript; □, loxiglumide; ▲, lorglumide; ∇, L-365,260; ●, devazepide.

thymidine; no such growth effects were observed with asynchronous cells. Specific culture conditions involving synchronisation of cells with thymidine and growth in serum-free medium have been reported previously to be important in revealing trophic effects of gastrin on gastric and colon cancer cells *in vitro* (Kusyk *et al.*, 1986; Watson *et al.*, 1988; Guo *et al.*, 1990). In previous studies, ornithine decarboxylase activity, or $^{3}H$-thymidine or $^{75}Se$-selenomethionine incorporation were used as indices of responsiveness of AR4-2J cells to gastrin (Scemama *et al.*, 1989; Seva *et al.*, 1990; Watson *et al.*, 1991*a*). Interpretation of data obtained by the latter approach is frustrated by the fact that gastrin increases secretion, and presumably synthesis, of proteins such as the enzyme amylase from AR4-2J cells (Lambert *et al.*, 1991). We assessed cell numbers using the MTT assay which relies on the ability of viable cells to reduce tetrazolium to formazan. It is recognised that this assay might be influenced by a variety of factors independent of cell numbers, including chemical interference with the tetrazolium or cellular enzymes, or cellular metabolic conditions (Scudiero *et al.*, 1988). However, the MTT assay has been shown to provide a reproducible index of cell proliferation in comparison with assays of cell protein in over 30 human and other tumour cell lines (Alley *et al.*, 1988; Rubinstein *et al.*, 1990), and in our study we confirmed the MTT results with direct cell counts. Gastrin at $10^{-12}$ M increased the proliferation of AR4-2J cells and at this low concentration would not be expected to interact with CCK-A receptors, as these require gastrin to be present in the micromolar range. This conclusion is supported by the rank order of potency of the antagonists for displacement of $^{125}I$-hG17-1 binding which is consistent with interaction at gastrin/CCK-B receptors. Previous studies have provided further evidence for growth stimulation coupled to gastrin/CCK-B, and not CCK-A receptors in AR4-2J cells (Scemama *et al.*, 1989). The response in pancreatic AR4-2J tumour cells contrasts with that in the normal mouse pancreatic acinar cells in which the growth response to gastrin is reported to be mediated via the CCK-A receptor (Logsdon, 1987). Human pancreatic cancer cell growth is stimulated by CCK (Smith *et al.*, 1990*b*; 1991); the receptors mediating this response require evaluating. The sequence of events linking occupation of gastrin/CCK-B receptors to enhanced cell division has yet to be elucidated, but early events may include the activation of $Na^{+}/H^{+}$ exchange (Bastie *et al.*, 1988) and ornithine decarboxylase (Scemama *et al.*, 1989).

The inhibition of AR4-2J proliferation by the anti-gastrin Ig preparation is evidence for an autocrine role for gastrin-like peptides in the growth regulation of these cells. Similarly, Hoosein and co-workers (1988, 1990) demonstrated that a gastrin antiserum, as well as gastrin/CCK receptor antagonists, inhibited proliferation of six human colonic cancer cell lines and suggested that gastrin-like peptides may function as autocrine growth factors in these cells. In the present study, the reduction in cell growth in the presence of the anti-gastrin reagent was very dramatic with a maximal mean reduction in proliferation of 52%. This would imply that gastrin-like peptides are very important autocrine growth factors for these cells and would explain the relatively small growth response to exogenous gastrin. An autocrine regulatory role for gastrin is supported by the presence of gastrin-like immunoreactivity in AR4-2J cells. The amount of gastrin-like immunoreactivity secreted by the cells was towards the detection limit of the assay, but within the range of concentrations of exogenous gastrin required to stimulate proliferation. Gastrin-like immunoreactivity has recently been reported in several human gastric and colonic tumour cell lines and this is secreted into the medium (Hoosein *et al.*, 1990; Watson *et al.*, 1991*b*). That this immunoreactivity reflects endogenous gastrin-like peptides has been confirmed by detection and sequencing of gastrin mRNA in these cells (Hoosein *et al.*, 1990; Baldwin *et al.*, 1990). The nature of the gastrin-like immunoreactivity in AR4-2J cells has yet to be elucidated.

All of the gastrin/CCK receptor antagonists tested inhibited AR4-2J proliferation over a 4 day period. It has been suggested by some investigators that proglumide-inhibition of cellular proliferation may be a result of non-specific effects rather than its ability to interact with gastrin receptors (Imdahl *et al.*, 1989; Singh *et al.*, 1987; Guo *et al.*, 1990). The concentrations of proglumide, benzotript, lorglumide and loxiglumide required for half maximal inhibition of AR4-2J cell growth were directly related to the concentrations required to displace 50% of specific $^{125}I$-hG17-1 binding to gastrin/CCK-B receptors, consistent with growth inhibitory actions mediated through gastrin/CCK-B receptor antagonism. However, these same antagonists also reduced proliferation of a cell line, HT-29, in which we were unable to demonstrate specific $^{125}I$-hG17-1 binding. This might be taken as evidence that the growth inhibitory effects of these antagonists are non-specific in nature. Alternatively, the inhibition of HT-29 proliferation by these compounds may indicate that gastrin receptors are expressed on HT-29 cells, but at levels too low to allow detection by $^{125}I$-hG17-1 binding. In addition, the inhibition of AR4-2J proliferation caused by the amino acid derivatives was partially reversed by gastrin, again consistent with involvement, at least in part, of gastrin receptors in the mechanism of action of these antagonists. In contrast, the two benzodiazepine derivatives (L-365,260 and devazepide) did not appear to inhibit AR4-2J cell growth by interaction with gastrin/CCK-B receptors. Much greater concentrations (several orders of magnitude) of these compounds were required to inhibit AR4-2J cell proliferation as compared with their ability to compete with $^{125}$I-hG17 binding. Inhibition of AR4-2J cell growth by these latter two agents was not reversible with exogenous gastrin. These results with L-365,260 and devazepide are similar to those recently reported by Thumwood *et al.* (1991) who found that devazepide in high concentrations inhibited the growth of a variety of cell lines but that this inhibition could not be reversed by addition of gastrin or CCK. These workers failed to observed inhibition of cell growth with the specific gastrin CCK-B receptor antagonist L-365,260, probably a reflection of the concentrations investigated, which were lower than in the present study. Devazepide and L-365,260 have gained in popularity as tools to define gastrin/CCK receptor sub-types since their introduction as *specific* CCK-A and CCK-B receptor antagonists. The results from the present study would urge caution in their unqualified use for such studies.

A hypothesis to account for the results obtained in the present study with L-365,260 and devazepide would be the existence of two gastrin/CCK-B receptor sub-types on AR4-2J cells; one major sub-type which recognises all six of the antagonists with varying affinity but which is not linked to cell growth processes and a second minor receptor sub-type with low affinity for L-365,260 and devazepide, but similar affinity to the first receptor sub-type for the other antagonists and which is responsible for the growth effects of gastrin on AR4-2J cells. In this hypothesis, it would be postulated that L-365,260 and devazepide decrease AR4-2J proliferation by a receptor-independent mechanism, consistent with the failure of exogenous gastrin to reverse the inhibition. At this time, the existence of such receptor sub-types on AR4-2J cells is speculation. The existence of distinct gastrin and CCK-B, as well as CCK-A, receptors has been argued by others on the basis of agonist studies (Jensen *et al.*, 1989), although previous studies with the benzodiazepine-derived antagonists have suggested that gastrin and CCK-B receptors are equivalent (Freidinger *et al.*, 1989). Functional studies using L-365,260 report it to have a lower affinity than predicted from binding studies in gastric mucosa; estimates of binding affinity may reflect interaction with CCK-B receptors, while the biological response may be a result of interaction with gastrin receptors (Patel & Spraggs, 1992). Three types of gastrin/CCK receptors have been discerned on guinea-pig smooth muscle cells from protection studies (Grider & Makhlouf, 1990). To extrapolate to the present results, on the basis of the studies with antagonists, gastrin receptor binding displacement may be an indication of CCK-B receptor affinity while inhibition of cell proliferation may be a consequence of gastrin receptor antagonism. However, the rank order for displacement of gastrin binding by agonists in AR4-2J cells suggested that gastrin binding is mainly a reflection of gastrin, rather than CCK-B, receptors (Scemama *et al.*, 1989). Obviously, further investigation is required to clarify which receptor sub-types reflect which functions.

In conclusion, we have shown a direct trophic effect of exogenous gastrin on AR4-2J rat pancreatic tumour cells. Evidence was also found for gastrin acting as an autocrine growth regulatory factor in these cells; significant concentrations of gastrin-like immunoreactivity were determined in lysates of AR4-2J cells and their proliferation was inhibited by gastrin/CCK receptor antagonists and by an anti-gastrin Ig preparation. From this study it would appear that gastrin receptor antagonists, or compounds which interfere with gastrin release by tumour cells, warrant further investigation as possible anti-tumour agents for cancers which express gastrin receptors. In this respect gastrin, rather than CCK-B, receptor antagonists would appear to be more promising.

This work was supported by a grant from Yamanouchi Pharmaceutical Co. Ltd., Tokyo, in collaboration with Ferring Research Institute, Southampton. We thank Dr R. Freidinger, Merck Sharp & Dohme, and Dr L. Rovati and Prof I. Setnikar, Rotta, for gifts of gastrin/CCK antagonists. Dr S. Watson, Nottingham is thanked for advice on the gastrin receptor binding protocol.

### References

ALLEY, M.C., SCUDIERO, D.A., MONKS, A., HURSEY, M.L., CZERWINSKI, M.J., FINE, D.L., ABBOTT, B.J., MAYO, J.G., SHOEMAKER, R.H. & BOYD, M.R. (1988). Feasibility of drug screening with panels of human tumor cell lines using a microculture tetrazolium assay. *Cancer Res.*, **48**, 589–601.

BALAS, D., SENEGAS-BALAS, F., PRADAYROL, L., VAYSSETTE, J., BERTRAND, C. & RIBET, A. (1985). Long term comparative effect of cholecystokinin and gastrin on mouse stomach, antrum, intestine and exocrine pancreas. *Am. J. Anat.*, **174**, 27–43.

BALDWIN, G.S., CASEY, A., MANTAMADIOTIS, T., MCBRIDE, K., SIZELAND, A.M. & THUMWOOD, C.M. (1990). PCR cloning and sequence of gastrin mRNA from carcinoma cell lines. *Biochem. Biophys. Res. Comm.*, **170**, 691–697.

BASTIE, M.J., DELVAUX, M., DUFRESNE, M., SAUNIER-BLACHE, J.S., VAYSSE, N. & RIBET, A. (1988). Distinct activation of $Na^+$-$H^+$ exchange by gastrin and CCK peptide in acini from guinea-pig. *Am. J. Physiol.*, **254**, G25–G32.

BEAUCHAMP, R.D., TOWNSEND, C.M., SINGH, P., GLASS, E.J. & THOMPSON, J.C. (1985). Proglumide, a gastrin receptor antagonist, inhibits growth of colon cancer and enhances survival in mice. *Ann. Surg.*, **202**, 303–309.

CREUTZFELDT, W. (1988). The achlorhydria-carcinoid sequence: role of gastrin. *Digestion*, **39**, 61–79.

ENOCHS, M.R. & JOHNSON, L.R. (1977). Trophic effects of gastrointestinal hormones: physiological implications. *Fed. Proc.*, **36**, 1942–1947.

FREIDINGER, R.M., BOCK, M.G., DIPARDO, R.M., EVANS, B.E., RITTLE, K.E., WHITTER, W.L., VEBER, D.F., ANDERSON, P.S., CHANG, R.S.L. & LOTTI, V.J. (1989). Development of selective non-peptide $CCK_A$ and $CCK_B$/gastrin receptor antagonists. In: *The Neuropeptide Cholecystokinin (CCK): Anatomy and Biochemistry, Receptors, Pharmacology and Physiology*, Hughes, J., Dockray, G.J. & Woodruff, G.N. (eds) Ellis Horwood: Chichester, pp. 123–132.

GRIDER, J.R. & MAKHLOUF, G.M. (1990). Distinct receptors for cholecystokinin and gastrin on muscle cells of stomach and gallbladder. *Am. J. Physiol.*, **259**, G184–G190.

GUO, Y.-S., BAIJAL, M., JIN, G.-F., THOMPSON, J.C., TOWNSEND, C.M. & SINGH, P. (1990). Growth-promoting effects of gastrin on mouse colon cancer cells *in vitro*: absence of autocrine effects. *In Vitro Cell. Dev. Biol.*, **26**, 871–877.

HOOSEIN, N.M., KIENER, P.A., CURRY, R.C. & BRATTAIN, M.G. (1990). Evidence for autocrine growth stimulation of cultured colon tumor cells by a gastrin/cholecytsokinin-like peptide. *Exp. Cell Res.*, **186**, 15–21.

HOOSEIN, N.M., KIENER, P.A., CURRY, R.C., ROVATI, L.C., MCGILBRA, D.K. & BRATTAIN, M.G. (1988). Antiproliferative effects of gastrin receptor antagonists and antibodies to gastrin on human colon carcinoma cell lines. *Cancer Res.*, **48**, 7179–7183.

HOWATSON, A.G. & CARTER, D.C. (1985). Pancreatic carcinogenesis: enhancement by cholecystokinin in the hamster-nitrosamine model. *Br. J. Cancer*, **51**, 107–114.

IMDAHL, A., EGGSTEIN, ST, CRONE, C. & FARTHMANN, E.H. (1989). Growth of colorectal carcinoma cells: regulation in vitro by gastrin, pentagastrin and the gastrin-receptor antagonist proglumide. *J. Cancer Res. Clin. Oncol.*, **115**, 388–392.

JENSEN, R.T., VON SCHRENCK, T., YU, D., WANK, S.A. & GARDNER, J.D (1989). Pancreatic cholecystokinin (CCK) receptors: comparison with other classes of CCK receptors. In *The Neuropeptide Cholecystokinin (CCK): Anatomy and Biochemistry, Receptors, Pharmacology and Physiology*, Hughes, J., Dockray, G.J. & Woodruff, G.N. (eds), Ellis Horwood: Chichester, pp. 150–162.

JESSOP, N.W. & HAY, R.J. (1980). Characteristics of two rat pancreatic exocrine cell lines derived from transplantable tumors. *In Vitro*, **16**, 212.

KUSYK, C.J., MCNIEL, N.O. & JOHNSON, L.R. (1986). Stimulation of growth of a colon cancer cell line by gastrin. *Am. J. Physiol.*, **251**, G597–G601.

LAMBERT, M., BUI, N.D. & CHRISTOPHE, J. (1991). Functional and molecular characterization of CCK receptors in the rat pancreatic acinar cell line AR4-2J. *Regul. Pept.*, **32**, 151–167.

LOGSDON, C.D. (1987). Effects of calcium mediated secretagogues on the growth of pancreatic acinar cells *in vitro*. *Gut*, **28** (Suppl. 1), 117–120.

McGREGOR, D.B., JONES, R.D., KARLIN, D.A. & ROMSDAHL, M.M. (1982). Trophic effects of gastrin on colorectal neoplasms in the rat. *Ann. Surg.*, **195**, 219–223.

MOSMANN, T. (1983). Rapid colorimetric assay for cellular growth and survival: application to proliferation and cytotoxicity assays. *J. Immunol. Methods*, **65**, 55–63.

PATEL, M. & SPRAGGS, C.F. (1992). Studies of gastrin/cholecystokinin receptors using functional isolated preparations. In *Multiple Cholecystokinin Receptors in CNS*, Dourish, C.T., Cooper, S.J., Iversen, S.D. & Iversen, L.L. (eds), pp. 98–106. Oxford University Press: Oxford (in press).

RUBINSTEIN, L.V., SHOEMAKER, R.H., PAULL, K.D., SIMON, R.M., TOSINI, S., SKEHAN, P., SCUDIERO, D.A., MONKS, A. & BOYD, M.R. (1990). Comparison of *in vitro* anticancer-drug-screening data generated with a tetrazolium assay versus a protein assay against a diverse panel of human tumour cell lines. *J. Natl Cancer Inst.*, **82**, 1113–1118.

SCEMAMA, J.L., DE VRIES, L., PRADAYROL, L., SEVA, C., TRONCHERE, H. & VAYSSE, N. (1989). Cholecystokinin and gastrin peptides stimulate ODC activity in a rat pancreatic cell line. *Am. J. Physiol.*, **256**, G846–G850.

SCEMAMA, J.L., FOURMY, D., ZAHIDI, A., PRADAYROL, L., SUSINI, C. & RIBET, A. (1987). Characterization of gastrin receptors on a rat pancreatic acinar cell line (AR4-2J): a possible model for studying gastrin mediated cell growth and proliferation. *Gut*, **28** (Suppl. 1), 233–236.

SCUDIERO, D.A., SHOEMAKER, R.H., PAULL, K.D., MONKS, A., TIERNEY, S., NOFZIGER, T.H., CURRENS, M.J., SENIFF, D. & BOYD, M.R. (1988). Evaluation of a soluble tetrazolium/formazan assay for cell growth and drug sensitivity in culture using human and other tumor cell lines. *Cancer Res.*, **48**, 4827–4833.

SEVA, C., DE VRIES, L., SCEMAMA, J.L., SARFATI, P., NICOLET, T.G., PRADAYROL, L. & VAYSSE, N. (1990). Gastrin modulates growth of a rat acinar pancreatic cell line: receptor analysis and signal transduction. *Digestion*, **46** (Suppl. 2), 166–169.

SINGH, P., LE, S., BEAUCHAMP, D., TOWNSEND, C.M. & THOMPSON, J.C. (1987). Inhibition of pentagastrin-stimulated up-regulation of gastrin receptors and growth of mouse colon tumor *in vivo* by proglumide, a gastrin receptor antagonist. *Cancer Res.*, **47**, 5000–5004.

SINGH, P., WALKER, J.P., TOWNSEND, C.M. & THOMPSON, J.C. (1986). Role of gastrin and gastrin receptors on the growth of a transplantable mouse colon carcinoma (MC-26) in BALB/c mice. *Cancer Res.*, **46**, 1612–1616.

SIRINEK, K.R., LEVINE, B.A. & MOYER, M.P. (1985). Pentagastrin stimulates *in vitro* growth of normal and malignant human colon epithelial cells. *Am. J. Surg.*, **149**, 35–39.

SMITH, J.P. & SOLOMON, T.E. (1988). Effects of gastrin, proglumide and somatostatin on growth of human colon cancer. *Gastroenterology*. **95**, 1541–1548.

SMITH, J.P., KRAMER, S. & BAGHERI, S. (1990*a*). Effects of a high-fat diet and L364,718 on growth of human pancreas cancer. *Dig. Dis. Sci.*, **35**, 726–732.

SMITH, J.P., KRAMER, S. & SOLOMON, T.E. (1991). CCK stimulates growth of six human pancreatic cancer cell lines in serum-free medium. *Regul. Peptides*, **32**, 341–349.

SMITH, J.P., SOLOMON, T.E., BAGHERI, S. & KRAMER, S. (1990*b*). Cholecystokinin stimulates growth of human pancreatic adenocarcinoma SW-190. *Dig. Dis. Sci.*, **35**, 1377–1384.

THUMWOOD, C.M., HONG, J. & BALDWIN, G.S. (1991). Inhibition of cell proliferation by the cholecystokinin antagonist L-364,718. *Exp. Cell Res.*, **192**, 189–192.

UPP, J.R., SINGH, P., TOWNSEND, C.M. & THOMPSON, J.C. (1989). Clinical significance of gastrin receptors in human colon cancers. *Cancer Res.*, **49**, 488–492.

WATSON, S.A., DURRANT, L.G. & MORRIS, D.L. (1988). Growth-promoting action of gastrin on human colonic and gastric tumour cells cultured *in vitro*. *Br. J. Surg.*, **75**, 342–345.

WATSON, S., DURRANT, L. & MORRIS, D. (1989). Gastrin: growth enhancing effects on human gastric and colonic tumour cells. *Br. J. Cancer*, **59**, 554–558.

WATSON, S., DURRANT, L., ELSTON, P. & MORRIS, D. (1991*a*). Inhibitory effects of the gastrin receptor antagonist (L-365,260) on gastronintestinal tumor cells. *Cancer*, **68**, 1255–1260.

WATSON, S.A., DURRANT, L.G., WENCYK, P.M., WATSON, A.L. & MORRIS, D.L. (1991*b*). Intracellular gastrin in human gastrointestinal tumor cells. *J. Natl Cancer Inst.*, **83**, 866–871.

WINSETT, O.E., TOWNSEND, C.M., GLASS, E.J. & THOMPSON, J.C. (1986). Gastrin stimulates growth of colon cancer. *Surgery*, **99**, 302–307.

YASUI, W. & TAHARA, E. (1985). Effect of gastrin on gastric mucosal cyclic adenosine 3′:5′-monophosphate-dependent protein kinase activity in rat stomach carcinogenesis induced by *N*-methyl-*N*′-nitrosoguanidine. *Cancer Res.*, **45**, 4763–4767.